# Efficacy of fire needle on patients of facial spasm: A protocol for systematic review and meta analysis

**DOI:** 10.1097/MD.0000000000022731

**Published:** 2020-10-23

**Authors:** Zhiying Zhong, Jun Xiong, Lunbin Lu, Jun Chen, Genhua Tang, Siyuan Zhu, Xingchen Zhou, Han Guo

**Affiliations:** a The Affiliated Hospital of Jiangxi University of Traditional Chinese Medicine; b Jiangxi University of Traditional Chinese Medicine, Nanchang, China.

**Keywords:** effectiveness, facial spasm, fire needle therapy, meta-analysis, protocol, systematic review

## Abstract

**Background:**

Facial spasm causes a lot of troubles to patients daily life and seriously affects their mental and physical health. Relevant studies have shown that fire needle therapy has certain benefits for facial spasm, is an integral part of acupuncture therapy. However, there is no unanimous conclusion. The main purpose of our study is to measure whether fire needle therapy is effective for facial spasm.

**Methods:**

The following electronic databases will be searched for the collection of fire-needle related randomized controlled trials (RCTS) for facial spasm, including 4 English databases (Web of Science, the Cochrane Library, EMBASE, Pubmed) and 3 Chinese databases (Chinese National Knowledge Infrastructure [CNKI], Wanfang data, Chinese VIP Information). The cure rate and total effective rate are the main outcomes, while the intensity, frequency, recurrence rate and adverse events are the secondary outcomes. We will use Endnote software X9 for study selection, Review Manager software 5.4 and STATA 13.0 software for analysis and synthesis.

**Results:**

We will evaluate the efficacy of fire needles in the treatment of facial spasm in combination with current studies.

**Conclusion:**

The conclusion of this study will provide evidence for the efficacy of fire needle in the treatment of facial spasm.

**Trial registration number:**

INPLASY202080036.

## 1 Introduction

Facial spasm is a kind of neuromuscular disease, with paroxysmal, involuntary unilateral, occasionally bilateral facial muscle convulsion as the main characteristic, through the facial nerve (the seventh cranial nerve) innervation.^[1]^ This is a chronic progressive disease, usually occurring between the ages of 40 and 70,^[2]^ but it can also affect teenagers^[3]^ and is more common in women (2:1).^[4]^ Previous studies have shown that Asians had a relatively higher prevalence rate than whites.^[5,6]^ The usual cause of facial spasm is compression of the outlet or point of entry of the facial nerve root from the seventh brain stem to the auditory canal.^[7–12]^ Facial spasm has significant effects on the mental and physical health of patients, such as mood disorders, visual disorders or language disorders.^[13–15]^ For example, facial spasm might lead to drooping eyelids and visual problems, which can lead to difficulties in reading and working. In addition, involuntary facial spasms are associated with a risk of dysarthria.^[16]^ Also, it can cause a lot of social awkwardness. Facial spasms are usually progressive and very little self-limiting,^[17]^ so prompt treatment is necessary.

The treatment of facial spasm mainly includes oral medications, botulinum neurotoxin injections and surgical treatment.^[3]^ However, existing treatments for facial spasm is not ideal, for the reason of limited therapeutic effect,^[16]^ unwanted side effects,^[18]^ the repeated cycle of a step,^[19]^ immeasurable risk, and postoperative complications.^[20]^ As a result, patients are more likely to seek alternative therapies for facial spasms.^[21]^

As a treasure of Traditional Chinese medicine, fire needle therapy has been paid more and more attention all over the world. It has been widely used in clinical practice and achieved good results. In other neurovascular conflicts such as myofascitis and periarthritis of shoulder, the advantages of fire needle therapy have been proved. Some studies have shown that fire needle therapy is beneficial for facial spasm. ^[22,23]^ However, the efficacy of fire needle in the treatment of facial spasm remains unclear due to the lack of a comprehensive assessment of the available evidence. Therefore, our objective is to systematically synthesize all randomized controlled trials (RCTS) of fire needle therapy for facial spasm to provide evidence for clinical practice of facial spasm.

## 2 Methods

### 2.1 Study registration

The protocol of our study has been registered on the INPLASY, (registration number: INPLASY202080036: https://inplasy.com/inplasy-2020-8-0036/.) The protocols reporting is carried out in strict accordance with the preferred reporting items of the Protocol on Systems Review and Meta-Analysis (PRISMA-P) guidelines.

### 2.2 Eligibility criteria

#### 2.2.1 Type of study

We will include RCTS of patients with facial spasm treated with acupuncture.

#### 2.2.2 Type of participant

Participants in the study were diagnosed with facial spasm. On the basis of “Neurology“^[24]^ “Clinical Neurosurgery”^[25]^ and “Clinical Pain Therapy”,^[26]^ the diagnostic criteria for facial spasm include uncontrollable spasm on 1 side of the face, which is manifested as intermittent orbicularis oculi spasm in the early stage and gradually extends to the mouth, eventually affecting the whole face; the severity of cramps varies and can be aggravated by stress, fatigue, conversation, and so on; the spasm terminates when the patient is asleep; in addition, neurological tests showed no positive signs. There are no age, gender or race restrictions.

#### 2.2.3 Type of intervention

The purpose of this study was to investigate the clinical trial of fire needle therapy for facial spasm. Studies using fire needles in experimental groups will be included. If the efficacy of the fire needle cannot be determined in the combination therapy, the combination of the fire needle and other treatments will be excluded. Conventional acupuncture, electroacupuncture, ear acupuncture or pharmacologic therapy may be used in the control group.

#### 2.2.4 Types of outcome measurements

##### 2.2.4.1 Primary outcome

- The cure rate (number of participants who made a full recovery/total number of participants in this group);
- The total effective rate (number of participants who showed a positive response to therapy/total number of participants in this group).

##### 2.2.4.2 Secondary outcomes

- Changes in spasm intensity after treatment;
- Changes in spasm frequency after treatment;
- The recurrence rate (recurrence during the follow-up period the number of people/total number of participants in this group);
- Adverse events related intervention measures.

##### 2.2.4.3 Exclusion criteria

- Participants with the unclear diagnosis;
- Studies that did not use fire needle therapy as primary treatment in the intervention group;
- Data that cannot be extracted;
- Duplicated data;
- The studies where full text is unavailable.

### 2.3 Search methods for identification of studies

#### 2.3.1 Electronic data sources

PubMed, MEDLINE, EMBASE, Cochrane Library, China National Knowledge Infrastructure (CNKI), Wanfang data, Chinese Scientific Journals Database (VIP), and China biomedical literature database (CBM) will be searched to obtain eligible studies that have been published by 2020. A combination of various medical topic titles and non-grid terms will be used, including “facial spasm,” “semi-facial spasm,” “needle,” and “fire needle,” which will be searched individually or in combination. Language, population or national restrictions will not apply. Table 1 shows a draft search strategy using Pubmed, one of the electronic databases to be searched.

**Table 1:**
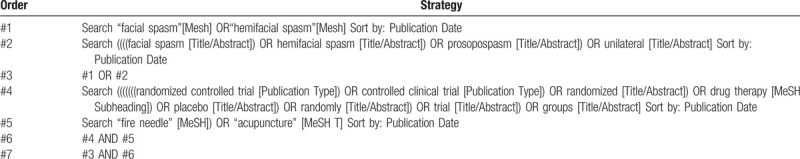
The search strategy for Pubmed.

#### 2.3.2 Other resources

A review or meta-analysis of relevant RCT systems will be conducted via electronic search. We also sift through relevant medical journals and journals to find literature that is not included in the electronic database.

### 2.4 Data collection

#### 2.4.1 Selection of studies

We will select the RCTs comparing the effecacy of fire needle in the treatment of facial spasm. Provisions that conform to one of the following will not include:

1. the duplicates,
2. the participants did not meet the diagnosis criteria of facial spasm or were not known,
3. not RCT studies,
4. Participants in the study did not receive a combination of fire acupuncture and conventional therapy as the primary intervention,
5. the intervention contains any other traditional Chinese medicine (TCM) therapy,
6. Incomplete data required.

**Figure 1:**
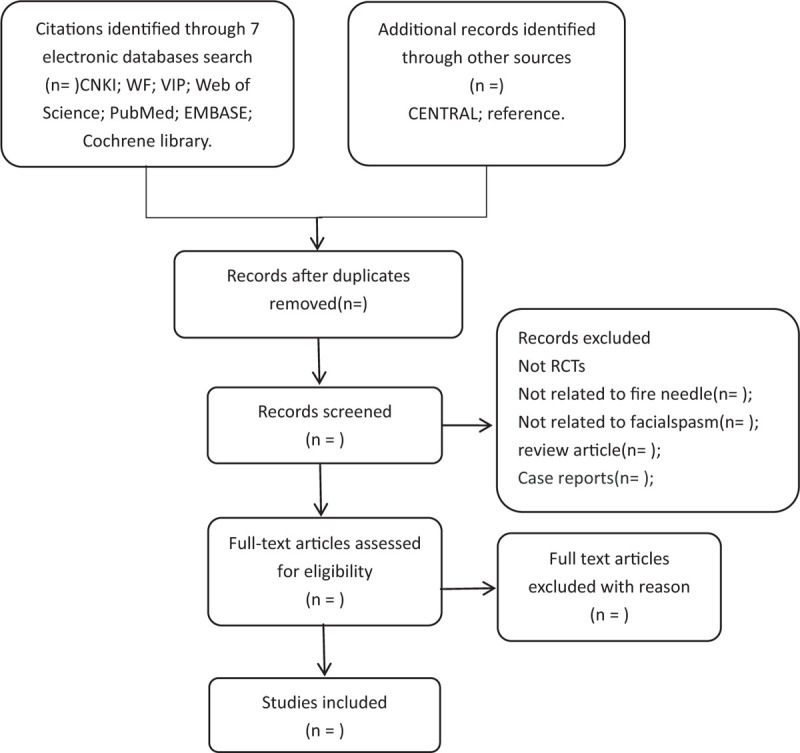
Flowchart of literature selection.

Whether these studies fit the bill will be assessed by 2 authors. If there are any objections in the contained articles, we will discuss and resolve them. The specific process of study selection will be shown in the flow chart of preferred reporting items in the Systematic Review and Meta-analysis (PRISMA) (Fig. 1).

#### 2.4.2 Data extraction and management

The other 2 authors will extract the data independently to fill out the pre- designed form. Any differences that occur during the data extraction process will be discussed. The following information will be extracted: study details (author, country, year of publication, multi-center, or single-center study), participant details (baseline data, diagnostic criteria), methods used (registration platform, sample size, blinded method), interventions, and results. For unreported data, we will contact the corresponding author.

#### 2.4.3 Assessment of risk of bias in included studies

According to the Cochrane Handbook, the risk of bias in each joined studies will be independently assessed to Systematic Reviews of Interventions.^[27]^ Rational judgment of risks will be evaluated and described from the following 7 aspects, including the generation of random sequences, the concealment of assignments, the blindness of participants and personnel, the blindness of outcome assessments, incomplete outcome data, selective reporting, and other biases. Risk will be divided into 3 levels: “low risk of bias,“ “high risk of bias,” and “unclear risk of bias.”

### 2.5 Data synthesis

If meta-analysis is possible, use the Review Manager software (RevMan5.4) and STATA 13.0 software for all data analysis. If significant statistical heterogeneity is detected, random-effects models are used for data synthesis. Otherwise, the data will be processed with a fixed effect model. If significant statistical heterogeneity exists, descriptive analysis is performed.

#### 2.5.1 Measures of treatment effect

For successive outcomes (the change in intensity after treatment, the change in frequency after treatment), we will use mean difference to evaluate the extracted data. For dichotomous outcomes (the cure rate, the total effective rate, the recurrence rate, and adverse events), we will analyze the rate ratio. For both continuous and dichotomous results, the confidence interval (CIs) is set to 95%.

#### 2.5.2 Management of missing data

If the information is insufficient or missing, the corresponding author will be contacted. If accurate data cannot be obtained after contacting the corresponding author, these studies will be excluded.

#### 2.5.3 Assessment of heterogeneity and subgroup analysis

We will conduct the qualitative analysis by comparing the characteristics of the included studies, and assess heterogeneity through the *I*^*2*^test and the *X*^*2*^test for quantitative analysis. If heterogeneity is evaluated as significant (*I*^*2*^ ≥ 50%) and the trials included are adequate, we will conduct a subgroup analysis to explore the potential source of the heterogeneity based on the differences in participant characteristics, interventions, controls, and outcome measures.

#### 2.5.4 Assessment of reporting biases

Funnel plots were selected to assess potential publication bias when the number of included RCTS ≥10.Otherwise, we will use the STATA 13.0 software to execute the Egger tests.

#### 2.5.5 Sensitivity analysis

Based on the risk of bias, insufficient data, and sample size, we will perform a sensitivity analysis to evaluate the robustness if significant statistical heterogeneity existed.

### 2.6 Grading the quality of evidence

Based on the proposed Grading of Recommendations Assessment, Development, and Evaluation,^[28]^ we will evaluate each outcome from 5 aspects of evidence quality (restrictive study design, inconsistencies, inaccuracies, inaccuracy, publication, and bias) and rank at 4 quality levels (very low, low risk, medium risk, and high).

### 2.7 Ethics and dissemination

Considering that our study is not related to individual patient data, ethical approval is not necessary. Our findings will be presented in a peer-reviewed journal or at a related meeting to assess the significance of fire needle therapy in patients diagnosed with facial spasm.

## 3 Discussion

Facial spasm brings a lot of troubles to patients work and life, seriously affects their mental and physical health. Currently, the best treatments for facial spasms are still controversial. They are accompanied by more or less adverse events and side effects, and do not completely eliminate the symptoms and harms of facial spasms. Therefore, it is urgent to seek alternative therapies with high efficiency and low side effects.

Fire needle therapy may be an effective treatment for facial spasm by regulating humoral immunity and autocellular immunity. However, there is no clear conclusion. In strict compliance with the Cochrane Handbook for Systematic Reviews of Interventions, we will conduct a systematic review and meta-analysis on the basis of existing RCTs to evaluate the efficacy of fire needle therapy for facial spasm, mainly to provide the basis for clinical practice and future research.

However, the evidence for better therapeutic effect of fire needle is not sufficient, and its mechanism of action is still unclear. Therefore, a systematic review and meta-analysis of the existing literature were carried out to objectively evaluate the clinical efficacy of fire needle in the treatment of facial spasm.

To our knowledge, this will be the first systematic review and meta-analysis of fire needle therapy for facial spasm. First of all, the conclusions of this review will provide objective statistical data for future studies of the fire needle. Secondly, it can provide reliable reference for doctors to use fire needle to treat facial spasm. Finally, effective alternative therapies can be provided to patients to ease the public health burden.

## Author Contributions

All authors have read and approved the publication of the protocol.

**Conceptualization:** Zhiying Zhong, Jun Xiong.

**Data curation:** Zhiying Zhong, Lunbin Lu, Jun Chen, Genhua Tang, Siyuan Zhu, Han Guo.

**Formal analysis:** Zhiying Zhong, Siyuan Zhu, Lunbin Lu.

**Investigation:** Jun Xiong, Zhiying Zhong.

**Methodology:** Zhiying Zhong, Genhua Tang, Lunbin Lu, Jun Chen, Siyuan Zhu.

**Software:** Jun Xiong, Siyuan Zhu.

**Supervision:** Jun Xiong, Genhua Tang.

**Writing – original draft:** Jun Xiong, Zhiying Zhong, Lunbin Lu, Han Guo.

**Writing – review & editing:** Jun Xiong, Siyuan Zhu, Genhua Tang, Lunbin Lu, Jun Chen.

## References

[1] Chopade TR Bollu PC Hemifacial Spasm. 2020 Aug 16. In: StatPearls [Internet]. Treasure Island (FL): StatPearls Publishing; 2020 Jan. PMID: 30252364. 30252364

[2] Czyz CN Burns JA Petrie TP Long-term botulinum toxin treatment of benign essential blepharospasm, hemifacial spasm, and Meige syndrome. Am J Ophthalmol 2013;156:173–7. 23541393 10.1016/j.ajo.2013.02.001

[3] Abbruzzese G Berardelli A Defazio G Hemifacial spasm. Handb Clin Neurol 2011;100:675–80. 21496615 10.1016/B978-0-444-52014-2.00048-3

[4] Auger RG Whisnant JP Hemifacial spasm in rochester and olmsted county, minnesota, 1960 to 1984. Arch Neurol 1990;47:1233. 2241620 10.1001/archneur.1990.00530110095023

[5] Poungvarin N Devahastin V Viriyavejakul A Treatment of various movement disorders with botulinum A toxin injection: an experience of 900 patients. J Med Assoc Thai 1995;78:281–8. 7561552

[6] Wu Y Davidson AL Pan T Asian over-representation among patients with hemifacial spasm compared to patients with cranial– cervical dystonia. J Neurol Sci 2010;298:61–3. 20864122 10.1016/j.jns.2010.08.017

[7] Eckman PB Kramer RA Altrocchi PH Hemifacial spasm. Arch Neurol 1971;25:81–7. 5316997 10.1001/archneur.1971.00490010091012

[8] Tan EK Chan LL Clinico-radiologic correlation in unilateral and bilateral hemifacial spasm. J Neurol Sci 2004;222:59–64. 15240197 10.1016/j.jns.2004.04.004

[9] Jitpimolmard S Tiamkao S Laopaiboon M Long term results of botulinum toxin type A (Dysport) in the treatment of hemifacial spasm: a report of 175 cases. J Neurol Neurosurg Psychiatry 1998;64:751–7. 9647304 10.1136/jnnp.64.6.751 PMC2170133

[10] Iwakuma T Matsumoto A Nakamura N Hemifacial spasm. Comparison of three different operative procedures in 110 patients. J Neurosurg 1982;57:753. 7143057 10.3171/jns.1982.57.6.0753

[11] Jannetta PJ Abbasy M Maroon JC Etiology and definitive microsurgical treatment of hemifacial spasm. Operative techniques and results in 47 patients. J Neurosurg 1977;47:321–8. 894338 10.3171/jns.1977.47.3.0321

[12] Kenney C Jankovic J Botulinum toxin in the treatment of blepharospasm and hemifacial spasm. J Neural Transm 2008;115:585–91. 17558461 10.1007/s00702-007-0768-7

[13] Tan EK Fook-Chong S Lum SY Botulinum toxin improves quality of life in hemifacial spasm: validation of a questionnaire (HFS-30). J Neurol Sci 2004;219:151–5. 15050451 10.1016/j.jns.2004.01.010

[14] Rudzińska M Wójcik M Szczudlik A Hemifacial spasm non-motor and motor-related symptoms and their response to botulinum toxin therapy. J Neural Transm 2010;117:765–72. 20467763 10.1007/s00702-010-0416-5

[15] Tan EK Fook-Chong S Lum SY Case-control study of anxiety symptoms in hemifacial spasm. Mov Disord 2006;21:2145–9. 17044052 10.1002/mds.21150

[16] Wang A Jankovic J Hemifacial spasm: clinical findings and treatment. Muscle Nerve 1998;21:1740–7. 9843077 10.1002/(sici)1097-4598(199812)21:12<1740::aid-mus17>3.0.co;2-v

[17] Leone JAM Jr Dhillon TS Treatment choices of 119 patients with hemifacial spasm over 11 years. Clin Neurol Neurosurg 1996;98:213. 8884091 10.1016/0303-8467(96)00025-x

[18] Kemp LW Reich SG Hemifacial Spasm. Curr Treat Options Neurol 2004;6:175–9. 15043800 10.1007/s11940-004-0009-4

[19] Tan NC Chan LL Tan EK Hemifacial spasm and involuntary facial movements. QJM 2002;95:493. 12145388 10.1093/qjmed/95.8.493

[20] Mclaughlin MR Jannetta PJ Clyde BL Microvascular decompression of cranial nerves: lessons learned after 4400 operations. J Neurosurg 1999;90:1–8. 10.3171/jns.1999.90.1.0001 10413149

[21] Kilduff CL Casswell EJ Salam T Use of alleviating maneuvers for periocular facial dystonias. JAMA Ophthalmol 2016;134:1247. 27606483 10.1001/jamaophthalmol.2016.3277

[22] Zhang L Zhao L Bai Y Comparative observation of the efficacy on facial spasm among different therapies. Zhongguo Zhen Jiu 2017;37:35–8. 29231320 10.13703/j.0255-2930.2017.01.008

[23] Qian J Xu W Clinical observation of fire needle for facial spasm. Zhongguo Zhen Jiu 2015;35:1221–4. 26964161

[24] Jiang W Jianping J Liying C Neurology (Chinese). Beijing: People's Medical Publishing House; 2005.

[25] Chengyuan W Clinical neurosurgery (Chinese). Beijing: People's Medical Publishing House; 2007.

[26] Zhonglian L Jianxiong A Jiaxiang N Clinical Pain Therapy (Chinese). Tianjin: Tianjin Science & Technology Press; 1994.

[27] Higgins JP Altman DG Assessing Risk of Bias in Included Studies. 2011;Chichester, UK: John Wiley & Sons Ltd, 187–241.

[28] Balshem H Helfand M Schünemann HJ GRADE guidelines: 3. Rating the quality of evidence. J Clin Epidemiol 2011;64:401–6. 21208779 10.1016/j.jclinepi.2010.07.015

